# Adult T-cell lymphoma/leukemia following infective dermatitis in an adult with HTLV-1 infection: a case report^☆^

**DOI:** 10.1016/j.abd.2025.501151

**Published:** 2025-07-01

**Authors:** Denis Miyashiro, Tatiane Assone, Augusto César Penalva de Oliveira, Sabri Saeed Mohammed Ahmed Al-Sanabani, José Antonio Sanches, Jorge Casseb

**Affiliations:** a Department of Dermatology, Faculty of Medicine, Universidade de São Paulo, São Paulo, SP, Brazil; b Laboratory of Investigation in Dermatology and Immunodeficiencies, Department of Dermatology, Faculty of Medicine, Universidade de São Paulo, São Paulo, SP, Brazil; c Instituto de Doenças Infecciosas “Emílio Ribas”, São Paulo, SP, Brazil

*Dear Editor,*

Human T-lymphotropic Virus Type-1 (HTLV-1) was the first retrovirus directly linked to cancer development in humans. It exhibits CD4+ T-cell tropism, resulting in cell cycle acceleration, formation of immortal CD4+ and CD8+ clones, and exaggerated immune response.^1^ It is estimated that 10 million people worldwide are infected with HTLV-1, Brazil being the endemic country with the largest absolute number of infected individuals (almost one million).^2^ Diseases associated with HTLV-1 include Adult T-cell Lymphoma/Leukemia (ATLL), HTLV-1-Associated Myelopathy (HAM), Sjogren’s syndrome, uveitis, thyroiditis, pneumonitis, arthritis, polymyositis, Infective Dermatitis (ID), other skin manifestations, urinary symptoms, erectile dysfunction, and periodontal disease.^3^, ^4^, ^5^ Infective dermatitis may be associated with HAM in up to 50% of cases.^6^

ATLL has five clinical forms: acute, lymphomatous, chronic, smoldering, and primary cutaneous tumoral.^7^ Infective dermatitis is a chronic relapsing disorder that affects children, with few reports of late-onset disease, and rare cases progressing to ATLL.^7^ We describe a case of adult-onset ID and ATLL.

A 71-year-old woman infected with HTLV-1 for 22 years presented xerosis and generalized pruritus on the whole body for 12 months. She was diagnosed with atopic dermatitis and was treated unsuccessfully with topical and systemic steroids for eight months before she was evaluated in our department. She presented erythematous-exudative plaques associated with xerosis on the limbs (Fig. 1A). She reported weight loss and night sweats, but no neurological symptoms. Skin biopsy of an exudative plaque revealed hyperparakeratosis, spongiosis, exocytosis of lymphocytes, and superficial perivascular infiltrate, and a diagnosis of ID was made. She also presented multiple infiltrated papules and purpuric lesions on the trunk (Fig. 1B‒C), and histopathology revealed infiltration by atypical CD4+CD7- T-cells, compatible with ATLL (Fig. 2). She had no lymph node or visceral involvement. HTLV-1 proviral load was 189copies/10^4^ Peripheral Blood Mononuclear Cells (PBMCs) and T-cell proliferation assay revealed 1243 counts/minute, ten times higher than HTLV-1-negative control. Immunophenotyping showed reduced CD3 intensity and 79% of abnormal CD4+CD26- cells (Fig. 3). Polymerase chain reaction of rearranged γT-cell receptor gene revealed monoclonal expansion of T-cells in the blood (Fig. 4). Diagnosis of unfavorable chronic ATLL was made. She received intramuscular betamethasone and oral antihistamines with partial response. After the recurrence of symptoms, zidovudine (400 mg/day) and pulse therapy with methylprednisolone three times every 45 days were started, resulting in marked improvement of ID and ATLL skin lesions and blood alterations. During follow-up, she developed bone marrow involvement and died after four years of diagnosis of ID and ATLL.

**Figure 1:**
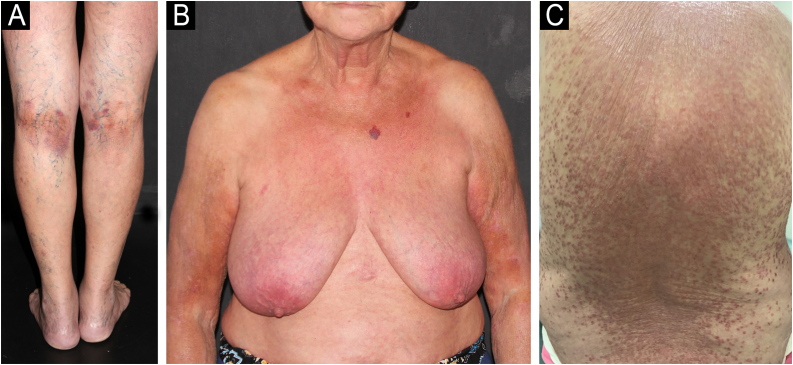
Erythematous and purpuric plaques on the popliteal fossae (A). Diffuse erythematous rash on the trunk and upper limbs (B). Multiple infiltrated papules on the back (C).

**Figure 2:**
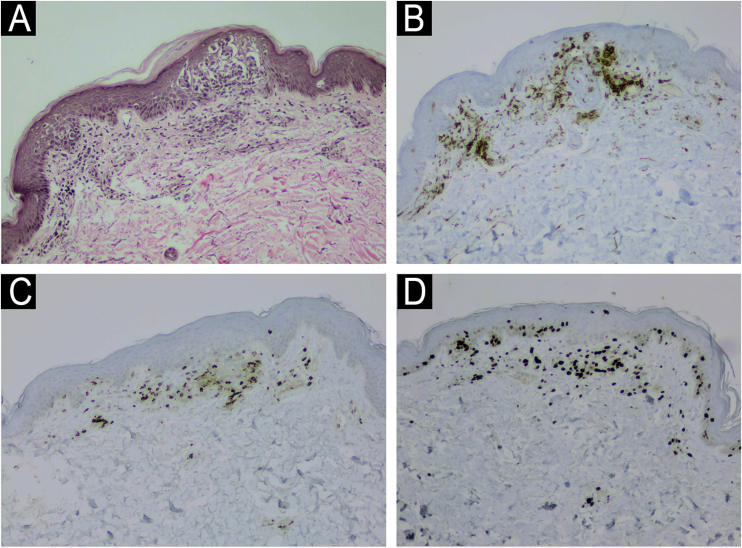
Histology of ATLL lesion showing exocytosis of lymphocytes with aggregates of atypical cells on the epidermis, and perivascular atypical lymphocytes on the superficial dermis (A, Hematoxylin & eosin, ×100); CD4 positivity (B, ×100); partial loss of CD7 (C, ×100); Ki-67 of 60% (D, ×100).

**Figure 3:**
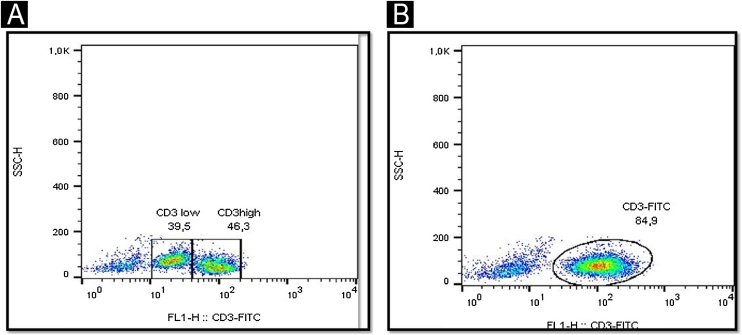
Immunophenotyping of lymphocytes in peripheral blood by flow cytometry showing lower CD3 intensity in abnormal T-cells (A) compared to a normal control (B).

**Figure 4:**
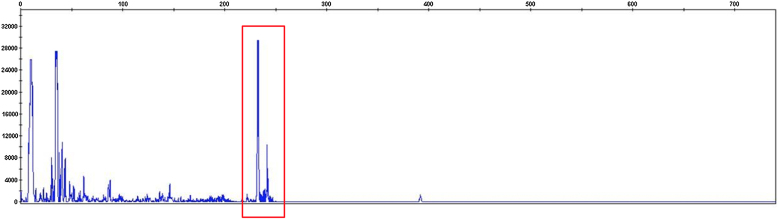
T-cell receptor gene rearrangement analysis show monoclonality with two differently sized gene rearrangements (red box).

Infective dermatitis is the primary pediatric manifestation in vertically infected HTLV-1 patients and is rare in adults.^5^, ^8^ Average age of onset is two years, and its prevalence decreases with age, probably due to maturation of the immune system.^9^ Clinical features comprise chronic erythematous-exudative eruptions affecting the scalp, retro auricular area, eyelids, the skin of paranasal sinus, axillae, neck, and groin.^8^ Lesions are aggravated by bacterial superinfections, particularly *Staphylococcus Aureus* (SA) and *Beta-Hemolytic Streptococcus* (BHS).^8^ Non-bacterial infections can also complicate the disease, such as cutaneous dermatophyte or Candida infections, and scabies.^8^

Development of ID has been associated with increased viral load, presence of HTLV-1 antibodies, and genetic predisposition.^8^ The possible pathogenesis would be the tax protein encoded by HTLV-1, which transactivates genes related to inflammatory cytokines (interferon-γ, tumor necrosis factor-alpha, interleukin-1, and interleukin-6).^9^ It has also been suggested that damage to the skin barrier due to HTLV-1-related dysregulation of epidermal proteinase and infection of Langerhans cells by HTLV-1 may lead to precarious modulation of immune responses in the skin and increased rate of SA and BHS infection.^10^ This chronic inflammation could induce malignant transformation of infected cells.^7^, ^8^ However, if ID in childhood predicts a higher risk for ATLL development in adulthood must be further investigated.

Prolonged antibiotic therapy is the best strategy for ID control. In this case, the patient received zidovudine, a nucleoside reverse transcriptase inhibitor, an effective therapy for ATLL as it exerts cytostatic effects by terminating DNA replication.^4^ We hypothesize that zidovudine may lower HTLV-1 replication in the skin and reduce inflammatory effects and immunological impairment of ID. In addition, steroid pulse therapy reduced cutaneous inflammation, resulting in significant improvement of ID and ATLL.

## Financial support

None declared.

## Authors' contributions

Denis Miyashiro: The study concept and design; data collection, or analysis and interpretation of data; writing of the manuscript or critical review of important intellectual content; effective participation in the research guidance; intellectual participation in the propaedeutic and/or therapeutic conduct of the studied case; critical review of the literature; final approval of the final version of the manuscript.

Tatiane Assone: The study concept and design; data collection, analysis, and interpretation of data; writing of the manuscript or critical review of important intellectual content; effective participation in the research guidance; critical review of the literature; final approval of the final version of the manuscript.

Augusto César Penalva de Oliveira: Data collection, or analysis and interpretation of data; writing of the manuscript or critical review of important intellectual content; intellectual participation in the propaedeutic and/or therapeutic conduct of the studied case; critical review of the literature; final approval of the final version of the manuscript.

Sabri Saeed Mohammed Ahmed Al-Sanabani: Data collection, or analysis and interpretation of data; writing of the manuscript or critical review of important intellectual content; effective participation in the research guidance; critical review of the literature; final approval of the final version of the manuscript.

José Antonio Sanches: Writing of the manuscript or critical review of important intellectual content; effective participation in the research guidance; final approval of the final version of the manuscript.

Jorge Casseb: The study concept and design; data collection, or analysis and interpretation of data; writing of the manuscript or critical review of important intellectual content; effective participation in the research guidance; intellectual participation in the propaedeutic and/or therapeutic conduct of the studied case; critical review of the literature; final approval of the final version of the manuscript.

## Conflicts of interest

None declared.
